# Promising Strategy of mPTP Modulation in Cancer Therapy: An Emerging Progress and Future Insight

**DOI:** 10.3390/ijms24065564

**Published:** 2023-03-14

**Authors:** Mohammad Waseem, Bi-Dar Wang

**Affiliations:** 1 Department of Pharmaceutical Sciences, School of Pharmacy and Health Professions, University of Maryland Eastern Shore, Princess Anne, MD 21853, USA; mwaseem@umes.edu; 2 Hormone Related Cancers Program, University of Maryland Greenebaum Comprehensive Cancer Center, Baltimore, MD 21201, USA

**Keywords:** mPTP, therapeutic targets, mPTP-mediated apoptosis, MOMP, oxidative stress

## Abstract

Cancer has been progressively a major global health concern. With this developing global concern, cancer determent is one of the most significant public health challenges of this era. To date, the scientific community undoubtedly highlights mitochondrial dysfunction as a hallmark of cancer cells. Permeabilization of the mitochondrial membranes has been implicated as the most considerable footprint in apoptosis-mediated cancer cell death. Under the condition of mitochondrial calcium overload, exclusively mediated by oxidative stress, an opening of a nonspecific channel with a well-defined diameter in mitochondrial membrane allows free exchange between the mitochondrial matrix and the extra mitochondrial cytosol of solutes and proteins up to 1.5 kDa. Such a channel/nonspecific pore is recognized as the mitochondrial permeability transition pore (mPTP). mPTP has been established for regulating apoptosis-mediated cancer cell death. It has been evident that mPTP is critically linked with the glycolytic enzyme hexokinase II to defend cellular death and reduce cytochrome c release. However, elevated mitochondrial Ca^2+^ loading, oxidative stress, and mitochondrial depolarization are critical factors leading to mPTP opening/activation. Although the exact mechanism underlying mPTP-mediated cell death remains elusive, mPTP-mediated apoptosis machinery has been considered as an important clamp and plays a critical role in the pathogenesis of several types of cancers. In this review, we focus on structure and regulation of the mPTP complex-mediated apoptosis mechanisms and follow with a comprehensive discussion addressing the development of novel mPTP-targeting drugs/molecules in cancer treatment.

## 1. Introduction

Mitochondria are considered as intracellular organelles crucial for energy generation and are also involved in several kinds of cellular damage and/or death including necrosis, apoptosis, and other impaired mechanisms via mitochondrial outer membrane permeabilization (MOMP) [1]. Mitochondria have been revealed to mediate the intrinsic apoptotic pathway provoked by the cytotoxic potency of different kinds of chemotherapeutic molecules [2]. These mitochondria-mediated intrinsic machinery could be triggered by different stimuli, such as high contents of cytoplasmic Ca^2+^, overproduction of reactive oxygen species (ROS), and other environmental factors such as UV damage [3,4,5]. Such stimuli activate apoptotic cascade, increase apoptotic protein expression, promote release of cytochrome c (Cyt C), and consequently promote/complete the eradication of the cells [6].

**Figure 1:**
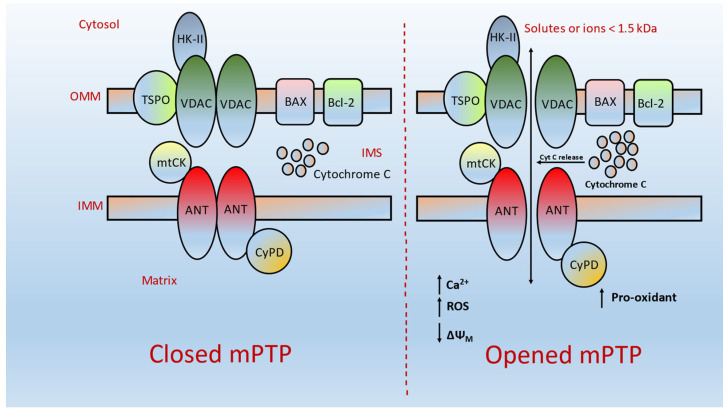
Schematic representation of the junction of mPTP at both opened and closed states. OMM, outer mitochondrial membrane; IMM, inner mitochondrial membrane; VDAC, voltage-dependent anion channel (mitochondrial porin); HK-II, hexokinase II; TSPO, translocator protein; ANT, adenine nucleotide transporter; CypD, cyclophilin D; Bcl-2, antiapoptotic protein; BAX, proapoptotic protein. Numerous stimuli such as pro-oxidant, enhanced intracellular Ca^2+^ levels, and overproduction of ROS are able to allow the free exchange of solutes and proteins smaller than 1.5 kDa between the mitochondrial matrix and the extra mitochondrial cytosol, ultimately causing mPTP opening.

Despite the fact the events underlying MOMP remain elusive, several protein complexes on mitochondrial membranes have been functionally linked with MOMP. In addition, the mitochondrial permeability transition (mPT) is believed to be critically involved in the mitochondria-mediated apoptosis [7,8]. mPT has been known as an alteration in the permeability of the inner mitochondrial membrane (IMM). During the past four decades, it was accepted that calcium overloaded/energized mitochondria could lead to substantial swelling episodes [9,10]. It has been suggested that swelling is due to a nonspecific permeabilization of the IMM via activation of Ca-sensitive phospholipases and thereby causes the production of fatty acids and lysophospholipids in the membrane. Nevertheless, pioneer studies by Haworth and Hunter [11] and later by Crompton and his coworkers [12] corroborated the evidence on increased permeability and showed the opening of a nonspecific channel with a well-defined diameter in the mitochondrial membrane that allows free exchange between the mitochondrial matrix and the extra mitochondrial cytosol of solutes and proteins smaller than 1.5 kDa. Later, this channel/pore was recognized as the mitochondrial permeability transition pore (mPTP) (Figure 1).

mPTP is a multiprotein channel complex found at the sites of mitochondrial membranes and is considered as a crucial regulator for the maintenance of the mitochondrial respiratory chain [13]. mPTP is predominantly composed of key proteins such as the voltage-dependent anion channel (VDAC) at the mitochondrial membrane (OMM), adenine nucleotide translocator 1 (ANT-1) at the inner mitochondrial membrane (IMM), and cyclophilin-D (Cyp-D) at the mitochondrial matrix platform [8]. Under normal physiological conditions, the mitochondrial inner membrane is impermeable, and only limited metabolites and/or ions can pass across the membranes. In healthy cells, mPTP normally remains closed and is triggered or opened upon external stimuli. It has been suggested that stimuli could induce the upregulation of both ANT-1 and Cyp-D, leading to mPTP opening and ROS production, ATP depletion and Cyt C release from the opened pore [14] (Figure 1). Notably, mPTP-mediated apoptosis machinery has been considered as an important clamp and plays a critical role in the pathogenesis of several kinds of cancers. It has been implicated that increased influxes of mitochondrial Ca^2+^ are generally predisposed to cellular death that could lead to intensifying mPTP-mediated cell invasion and proliferation in multiple cancer episodes. These events occur via a posttranslational scenario and/or the inhibitory action of several kinds of promalignant factors.

## 2. Cancer: Global Burden

Cancer has been recognized as a disorder arisen from genetic or epigenetic remodeling in somatic cells [15]. It is believed that cancer cells develop a degree of autonomy from alarming signals or stimuli, leading to unregulated growth and proliferation. If proliferation continues and transmits to the surrounding cells, it potentially leads to a more destructive/deadly episode. Indeed, almost 90% of cancer-associated mortality was observed owing to tumor spread, so-called metastasis [16,17,18].

Cancer is the second leading cause of mortality at the global level. Overall, the prevalence of cancer has exponentially increased; 1.9 million new cancer cases and 609,360 cancer deaths were projected to occur in 2022 in the United States [16]. According to global demographic investigations, about 0.42 billion new cases of cancer have been anticipated by 2025 every year, representing an expanding incidence of cancer in upcoming years. On this planet, new cancer was recorded as about 1,918,030 cases in 2022; overall prevalence in men was documented as about 983,160 and women about 934,870 cases. Globally, about 9.5 million casual cases or deaths were predicted in cancer. By 2022, for males the most prevalent and death cases in cancers are recorded as lung (1,435,943), prostate (1,414,259), and colorectal (1,065,960) cancer. For females, the most prevalent and death cases are recorded as breast (2,261,419), colorectal (865,630), and lung cancer, contributing 44.5% of the total number of new cases diagnosed in 2022.

## 3. Possible Functional Implications of mPTP in Cancer

**Figure 2:**
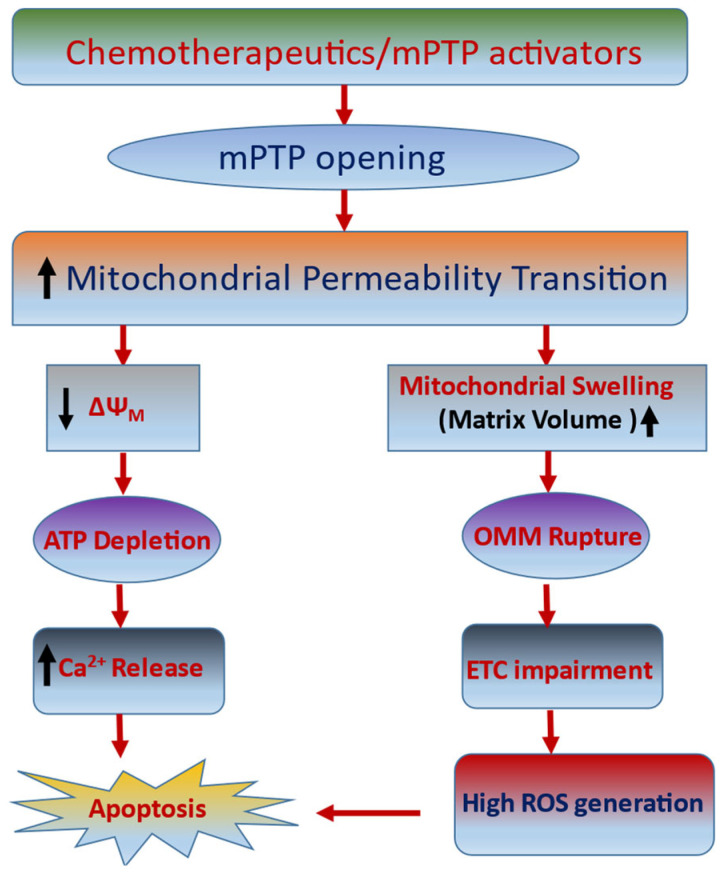
Plausible role of chemotherapeutics or modulators targeting mPTP in cancer. In cancer therapy, mPTP remains open, which causes the mitochondrial membrane potential to deteriorate followed by disruption of the OMM. This mitochondrial impairment results in the depletion of ATP generation, defective ETC, and enhanced Ca^2+^ flux, which leads to a massive generation of ROS and ultimately causes apoptosis.

The mPTP junction has emerged as a promising target for novel chemotherapeutics [16,19]. In this review, we focus on structure and regulation of the mPTP complex in apoptosis-mediated cancer cell death, followed by the development of novel drugs/compounds targeting mPTP for cancer therapy. The closure of mPTP establishes no chance of cellular collapse, which then constantly pushes to execute cellular blebbing or shrinkage due to the stimulation of caspases via the apoptosis mechanism. The greater capacity to enhance cellular death triggers such conditions and is an anchor for targeting malignant cell progression [17], and most chemotherapeutics function by directly or indirectly activating the apoptotic cascade in neoplastic cells. Accordingly, a deeper knowledge of mPTP structure and its regulation in cancer provides an appealing way to develop anticancer strategies. mPTP opening is triggered by both the Ca^2+^ overload in the mitochondrial matrix and the overproduction of ROS-mediated oxidative stress. It has been implicated that the opening of mPTP results in the increase of mitochondrial permeability that facilitates free fluxes of solutes (including water, molecules, and ions) into the mitochondrial matrix. In later stages, the opening of mPTP induces mitochondrial swelling, OMM rupture, and impairment in ETC, causing a massive release of ROS. Furthermore, the enhanced mitochondrial permeability also leads to loss of mitochondrial membrane potential (ΔΨ_M_), thereby causing a decrease of the cellular mitochondrial ATP and an increase of the intracellular Ca^2+^ levels. Consequently, the opening of mPTP induces the activation of endogenous apoptotic machinery, promoting chemotherapeutic-induced cancer cell death (Figure 2).

## 4. Mitochondrial Participation in Oxidative Stress and Cancer Development

It has been implicated that in healthy replicative eukaryotic cells, mitochondria have shown to be a crucial regulator for prominent cellular processes including proliferation, Ca^2+^ homeostasis, metabolic adaptation, and death. In addition, mitochondria are also the hub for extensive reactions, such as fatty acid oxidation (FAO), the tricarboxylic acid (TCA) cycle, and oxidative phosphorylation (OXPHOS). The primary steps are gluconeogenesis, ketogenesis, and heme biosynthesis [20,21]. In accordance with mitochondrial functionality, it is not surprising that mitochondrial impairment participates in a series of diseases, including cancer [22,23]. There have been more considerable mechanisms wherein mitochondria are involved in the progression of the malignant phenotype over the metabolic reprogramming of cancer cells. Primarily, it is ubiquitously investigated that most cancer diseases are critically linked with DNA mutations affecting mitochondrial functions, predominantly due to the modulation of subunits of the ETC. Interestingly, highlights of cancers including liver and prostate have been alarmingly linked with mutations in mitochondrial Complex I, and brain related cancers also harbor mutations in Complex II at mitochondrial sites [24,25,26].

Additionally, ROS-induced oxidative stress is a more prominent stimulus/signal for development and progression of cancer. It is evident that ROS is primarily produced in mitochondria for delivering superoxide as a by-product of oxidative phosphorylation. Mitochondrial ROS was shown with a massive production via the TCA cycle or in the ETC [24]. Unlike inducible reactions, ROS could also act as toxic species for cellular macromolecules. An enhanced-level ROS is frequently observed in cancer cells owing to the raised metabolic rates and modified antioxidant potential [27]. Multiple molecular events/changes leading to enhanced cell proliferation and anti-apoptosis are induced in mitochondria during tumorigenesis [28]. One such change is the hindrance of telomere erosion by an integral telomerase expression that establishes the maintenance of telomere length. It has been shown that telomerase reverses the translocation of transcriptase from nucleus to mitochondria under oxidative stress, restoring mitochondrial functionality and declining oxidative stress and therefore defending mitochondrial DNA and nuclear DNA from oxidative damage [29]. Many investigators have documented increased ROS production that then triggers protumorigenic signaling events, raising cellular proliferation and survival and stimulating DNA damage and genetic alterations [29,30,31,32]. Nevertheless, an increased level of ROS has been pointed out as enhancing antitumorigenic machinery by activating oxidative stress-mediated tumor cell death. Tumor cells evolve a cascade wherein an adjustment of high ROS was replenished by altered increased levels of antioxidant molecules to detoxify for maintaining protumorigenic signaling and resistance to apoptosis. Therefore, manipulation of ROS levels has been of great interest for potential cancer therapy. Taken together, mitochondrial dysfunction plays a critical role in cancer development/progression, and the molecular mechanism underlying mitochondria-mediated cancer clinical outcomes remains elusive.

## 5. Mitochondria as Model Organelle for Regulating Apoptosis Machinery

Each second, billions of cells encounter a well-controlled form of cell death called apoptosis. This cellular event plays a key role in maintaining our health and preventing us from cancer [33]. Cancer has been the result of accumulated gene mutations that result in uncontrolled cell proliferation. It has been well reported that apoptosis activity is frequently suppressed during tumorigenesis through different mechanisms. Likewise, matrix detachment of cells has an imperative effect for metastasis, which is then able to induce a critical stage of apoptosis called anoikis [34]. Such kinds of cell death events could play a key role in cancer therapeutic interventions. In fact, several anticancer therapies were developed based on provoking cell death/apoptosis via targeting/disturbing various mechanisms in cancer cells. In contrast, the anti-apoptosis phenotype observed in cancers play a critical role in promoting tumorigenesis and blocking therapeutic responses. However, apoptotic inhibition promotes cancer, and indeed, tumor cells are frequently more sensitive to cell death than is normal tissue.

In recent years, mitochondrion has been accepted as an excellent model for studying apoptotic cell death, and the molecular mechanism underlying mitochondrial-induced apoptosis is extensively investigated. The mitochondria-mediated apoptotic pathway has been characterized by caspase activation machinery via MOMP and ensuing a release of Cyt C from mitochondria to cytoplasm, thereby causing caspases activation that ultimately leads to cellular shrinkage and blebbing of the plasma membrane [35]. On the contrary, MOMP is controlled by the Bcl-2 family of apoptotic proteins. Such events play a key role in not only normal development and maintenance of tissue homeostasis but also human diseases including cancer [36,37]. An increase in mitochondrial membrane permeability is one of the considerable episodes in apoptotic death. However, the exact mechanism is yet to be clarified. It has also been confirmed that mitochondria-mediated apoptosis is a programmed event that requires some specified protein molecules to either directly or indirectly target cell death in cancer [38,39,40]. For the past two decades, development of therapeutic drugs and conventional chemotherapies has shown a considerable induction of apoptosis against different kinds of cancers including prostate cancer [41,42,43].

## 6. mPTP: A Gate for Apoptotic-Mediated Cancer Cell Death

It has been investigated that targeting these specialized mPTP components could lead to initiating cell death, and therefore mPTP is considered a key player in cancer. To develop novel therapeutic strategies targeting cancer cells, researchers have been exploring the possibility of modulating mPTP activity to inhibit tumor initiation and progression. In recent years, many scientific investigations on mPTP have been performed in cell lines derived from prostate [43], colon [44,45], cervical [46], osteosarcomas [47], pancreatic [48], and other kinds of cancers. In these cancer cell models, different drug and/or molecules were considered to evaluate mitochondrial integrity by examining mitochondrial membrane potential followed by the opening of mPTP due to enhanced mitochondrial oxygen consumption. It has been noted that reduced mitochondrial membrane potential could release Cyt C from mitochondria and activate a series of caspase events to establish the mechanistic model of apoptosis in cancer cells. mPTP has been considered as a molecular mechanism for increasing mitochondrial inner membrane permeability, allowing solutes, ions, or proteins up to 1.5 kDa to pass through [48,49]. mPTP consists of a voltage-dependent anion channel (VDAC) and the adenosine nucleotide transporter (ANT) located on the OMM and IMM, respectively. These two proteins were considered major components of the pore. These molecules and/or proteins are enveloped by a sequence of regulators, such as kinases including hexokinase II (HKII), glycogen synthase kinase 3β (GSK3β), and mitochondrial creatine kinase (mtCK); translocator protein (TSPO, formerly known as benzodiazepene receptor); cyclophillin D (CypD, a mitochondrial matrix protein found for peptidylprolyl cis-trans-isomerase activity); and members of the Bcl-2 family [49,50,51,52]. Precisely, the two proapoptotic candidates BAX and Bcl-2 were considered as homologous antagonists triggering the mPTP opening, as evident from the BAX- and BAK-knockout rodent models [53]. Previous studies have suggested that inhibiting BAX and BAX causes impairment of mitochondrial Ca^2+^ uptake, triggering mPTP opening by increasing the permeability of the OMM [54].

## 7. Targeting Mitochondria as Cancer Therapeutic Strategy

It has been well studied that a complex crosstalk occurs between mitochondria and ion channels, proteins and/or enzymes for ATP production, ROS deterioration, and proper supply of glucose at the metabolic platform [55,56]. These key functions are most prominent for cellular survival and growth. In cancer therapeutic intervention, targeting such kinds of channels, enzymes, and proteins linked with mitochondria could lead to a destructions of cancer cells [57]. In the context of ion channels/pores, there are specialized channels/pores that stay on the mitochondrial outer membrane and are responsible for the entrance of almost all the nutrients or ions and expel several apoptosis mediators. Such resident ion channels and/or pores on the mitochondrial outer membrane are considered as translocases that have the ability to transport protein, and the mitochondrial apoptosis-induced channel allows for the release of Cyt C and VDAC, able to transport metabolites. All these specialized channels play a key role in the formation of mPTP. This mPTP induction could further lead to apoptosis [40]. Overproduction of mitochondrial ROS and cytoplasmic Ca^2+^ stimulates mPTP opening, facilitating mitochondrial-mediated apoptosis. In addition, external stimuli-induced mPTP opening could lead to the translocation of Cyt C from the mitochondrial matrix into cytosol followed by strong binding with Apaf-1 and caspases to form a structure called apoptosome, thereby causing caspase-dependent apoptotic cell death pathways in cancer cells. Taken together, mPTP is considered to be a promising therapeutic target. Drugs/compounds targeting mPTP and inducing pore opening could lead to activation of the mPTP-mediated apoptosis cascade in mitochondria, representing a novel therapeutic strategy for cancer treatment. In the following sections, we discuss several therapeutic strategies by targeting mPTP components.

## 8. Targeting mPTP Components as Novel Cancer Interventions

### 8.1. Targeting VDAC and HK-II

**Figure 3:**
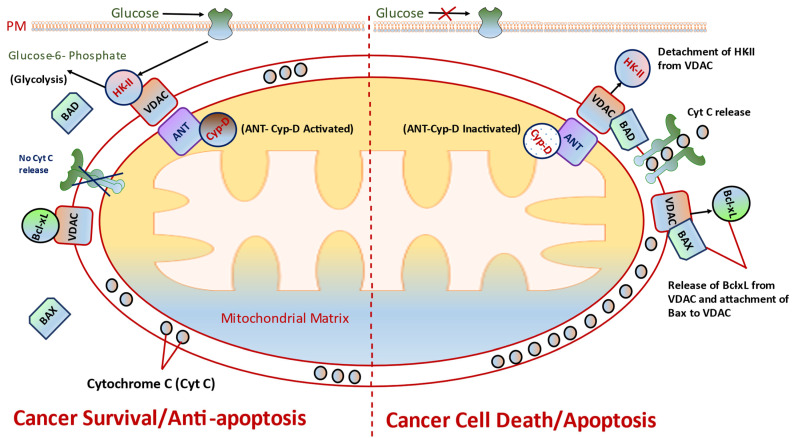
Mitochondrial-bound HKII is a well-considered player for providing glucose supplementation to cancer cells, thereby preventing tumor apoptosis. Targeting HKII or disrupting HKII–VDAC interaction will then initiate cell apoptosis. On left: cancer survival or antiapoptotic state persists in cancer cells within the critical conditions existing in tumor microenvironment. For instance, cytochrome c release, detachment of HKII from VDAC/ANT/CyD, and inactivation of BAX or BAD block the cell apoptotic cascade, and therefore no activation of the mPTP is allowed. On right: cancer cell death/apoptosis. HK-II is capable of detaching from VDAC, which blocks glucose supplementation and inhibits/decreases Bcl-2 proteins that in turn facilitates the switch-on of both apoptotic proteins BAX and BAD and alters both ANT and CyD, ultimately triggering mPTP opening and leading to the release of cytochrome c from the IMM to the cytosol.

VDAC is a critical component of mPTP and has been investigated as a frequently targeted hub for cancer therapy. VCAC has also been recognized as mitochondrial porins. Such porins have been implicated as facilitating the transport of key ions including Cl^−^, K^+^, Na^+^, and Ca^2+^; the delivery of ATP/ADP and NAD^+^/NADH; the movement of fatty acids and cholesterol; and the transfer of ROS between the inner mitochondrial membrane and cytosol [49]. In mammalian mitochondrial studies, three isoforms of VDAC have been determined: VDAC1, VDAC2, and VDAC3 [58]. Of these three isoforms, VDAC1 has been identified as being present in a more abundant form in mammalian mitochondria and has been shown to play a crucial role in mitochondrial apoptosis [59]. VDAC1 communicates with other apoptosis switching proteins, including members of the Bcl-2, antiapoptotic proteins such as Bcl-xL and apoptotic-repressing glycolytic enzymes such as HK, could activate cell survival [60]. Once the attachment and/or binding of apoptosis-mediated proteins to VDAC1 is hindered, this leads to an increase in the mitochondrial permeability transition (mPT) (Figure 3). This increase of mPT would further activate proapoptotic proteins (BAX and BAK) and thereby lead to moving across the mitochondrial outer membrane and entering into the cytosol wherein cellular apoptosis is executed [61]. Several compounds are recognized as proapoptotic molecules (cytotoxic agents) that are able to act (target/interfere) on either VDAC1 or HKII. This could inhibit their possible interactions or promote their detachment, inducing cell apoptosis [62,63]. Prezma et al. have synthesized VDAC1-based peptides and considered them as Antp-LP4 and N-terminal-Antp that could specifically destroy peripheral blood mononuclear cells pulled from patients with chronic lymphocytic leukemia. Interestingly, this did not show any impaired effects to normal cells obtained from healthy donors [64]. A similar research group has also formulated one new cell-penetrating peptide synthesized to improve target specificity and cell stability. This peptide was named R-Tf-D-LP4 and consists of a transferrin receptor that has a property to enhance the targeting of a peptide to cancer cells [65]. A study by Yuan et al. has implicated that the endostatin stimulates the opening of mPTP via targeting VDAC1, resulting in infiltration of cytochrome c from mitochondria into the cytosol and ultimately inducing cell apoptosis [66].

Cancer cells exhibit active aerobic glycolysis for their energy production in the form of ATP [67]. Most notably, hexokinase (HK) is a well-recognized enzyme for catalyzing the primary step of glycolysis, wherein glucose conversion to glucose-6-phosphate (G6P) was observed (Figure 3). There are four well-defined isoforms of hexokinase, including HKI, HKII, HKIII, and HKIV. Among all four isoforms, HKII has been characterized and known for high overexpression in cancer cells [68,69]. This kinase has the capability of binding with VDAC1 on the mitochondrial outer membrane, which leads to the interaction of VDAC1 with another mPTP component ANT on the mitochondrial inner membrane. This possible interaction facilitates the coupling of aerobic glycolysis that favors a strong relationship with OXPHOS in metabolic events. Such episodes could also show a rapid rate of glycolysis by prodding HKII to exchange ADP for ATP from the mitochondria [7,70]. Therefore, inhibiting HKII could lead to delaying or ceasing aerobic glycolysis, thereby causing cancer cell death. Therefore, HKII inhibition would not only hinder glycolysis but also repress the antiapoptosis and HKII–VDAC interaction in cancer (Figure 3).

### 8.2. Targeting CyP-D, a Key Regulator of HKII Binding to VDAC

Previous studies have indicated that CyP-D is overexpressed in many human cancers. Overexpression of CyP-D leads to repression of the apoptosis cascade [71]. Accumulating studies have demonstrated that the antiapoptotic regulation of CyP-D might be critically linked with the modeling of HKII-bound mitochondria [72,73]. This has been implicated as an inactivation of CyP-D with either cyclosporine A or siRNA knockdown of *PPID* (gene encoding *CyP*-D) to move out HKII from mitochondria. CyP-D is a well-defined mitochondrial matrix protein and is persistent for its alteration of HKII binding to VDAC. As discussed above, ANT at the IMM junction could play a role as mediator for such events [74]. Nevertheless, Chiara et al. have reported an opposite functional role of CyP-D as a proapoptotic protein [75]. Researchers also demonstrated that HKII detachment-provoked apoptosis might be linked with a deterioration of the interaction of CyP-D with ANT. Furthermore, inactivation of CyP-D could also promote an opening of the mPTP (Figure 3). Further studies are needed to clarify the possible role of CyP-D in apoptotic cascade. Disruption of HKII and other proapoptotic proteins to interact with VDAC is required for mPTP opening and is subsequently linked to the mPTP-mediated cell apoptosis. Moreover, the reduced form of CyP-D is involved in mPTP closing, while the oxidized form promotes mPTP opening [75,76]. Earlier reports have suggested an interaction between ATP synthase and the mPTP. The molecules that are considered to regulate mPTP opening/closing also have a binding affinity to ATP synthase and/or CypD. The CypD has more capacity to either directly or indirectly bind to ATP synthase and Bcl-2 family member Bcl-xL and could interact with the β-subunit of ATP synthase, leading to an enhanced efficiency of ATP production in the IMM. In addition, CypD is more capable of binding with ANT. This kind of interaction has reflected a more interesting fact. Therefore, ANT and ATP synthase in synthasomes also consist of mtCK, which binds to ANT [77]. One of the studies conducted by Bernardi et al. showed that mPTP forms at the mitochondrial membranes enclosing the dimers of ATP synthase [9].

### 8.3. Targets Bcl-2 Family Proteins

Bcl-2 is well recognized as a family member of antiapoptotic B-cell lymphoma 2, which is implicated in tumor episodes including initiation, progression, apoptosis, and/or programmed cellular death and response to chemotherapy [77,78]. Based on its molecular architecture and function, Bcl-2 has been indicated as both antiapoptotic and proapoptotic proteins. At the antiapoptotic platform, four Bcl-2 homology domains (BH1, BH2, BH3, and BH4) includes Bcl-2 and Bcl-2-related protein A1 (Bfl-1/A1), myeloid cell leukemia 1 (Mcl-1), and BCLB/Boo protein [78,79]. Likewise, the proapoptotic platform consists of two subfamilies: the multidomain proapoptotic ‘effectors’ (such as BAK, BAX and BOK) and those members that are known as ‘BH3-only proteins’ (BAD, BID, BIK, BIM, BMF, HRK, PUMA, and NOXA), as they possess only the short BH3 domain [80]. Such key proteins have great effects on mitochondrial permeabilization. It has been suggested that the activation of BAK and BAX could lead to oligomerization of BAK/BAX that further forms a pore, therefore enhancing mitochondrial permeability and ultimately leading to a sequence of events activating apoptosis cascade [81] (Figure 3).

## 9. Signaling Pathways Mediating mPTP Opening/Closing in Cancers

### 9.1. ERK Signaling

In recent years, a cluster of well-recognized oncogenes and/or tumor suppressor genes have shown the ability to alter Ca^2+^ homeostasis responsible for the regulation of mPT induction [13]. Convincingly, such factors are more capable of localizing on the mitochondrial membrane, possibly enhancing both selectivity and efficiency of their regulatory effect in AKT and two tumor suppressors, PML and PTEN. At this juncture, genes such as AKT, PML, and PTEN appear to exist in a complex that interacts with IP3, and this cascade has demonstrated a particular intention, as AKT and PTEN are both members of the PI3K pathway, which is an extensively investigated signaling mechanism and is considered as a key player for cancer therapies [82]. In the context of mitochondrial events, AKT has shown a great effect on mPTP activation via another mechanism that is indirectly associated to Ca^2+^ signaling [82,83,84,85].

In general, AKT has the propensity to induce the phosphorylation of GSK3β that leads to its inactivation. Notably, GSK3β kinase is a significant contributor for mPTP modulation. Its inactivation by AKT could enhance the association between HKII and VDAC, leading to an inhibition of mPTP opening and an increase in cancer cell survival [84]. Modulation and alteration in GSK3β activity have been investigated in different kinds of cancers [85,86,87]. Moreover, GSK3β has compelling effects, as it has the strength to act as a convergence factor, which allows survival-signaling molecules to interact with the mPTP. In addition, ERK/GSK3/CyP-D signaling has been implicated as a transduction axis and is considered the critical component for modulation of the mPTP opening/closing in tumor cells. In recent years, ERK has been delineated to be an integral activator of mitochondria in different kinds of cancers including prostate [88,89,90], osteosarcoma [91], endometrial [92], and Gastric cancer [93]. Several published reports have implicated that ERK activation inhibits the release of apoptogenic proteins, and on the contrary, the interference of ERK could also induce a profound reduction in cellular ATP, leading to loss of mitochondrial membrane potential and inducing cell apoptosis [90]. Additionally, ERK activation suppresses the GSK3-dependent phosphorylation of CyP-D (a mPTP regulator), altering mitochondrial permeability and blocking the opening of mPTP at the mitochondrial junction in cancer cells.

### 9.2. mTOR Signaling

mTOR, an extensively negative regulator of cellular autophagy, and plays crucial functions at the downstream of AKT signaling. mTOR is frequently upregulated in cancers, and mTOR overexpression could also block the mPTP opening to the same extent as activating AKT and inactivating GSK3β. In contrast, inhibition of mTOR interferes with the binding of HKII to VDAC, thereby activating cell apoptosis [94]. Therefore, kinase events are more necessarily activated in cancers and could retain HKII bound to the mitochondrial sites by blocking GSK3, hence guarding the closure of mPTP. Such a cascade may also affect the antiapoptotic phenotype of cancer cells and the progress of therapeutic resistance. In contrast, molecular strategy mediating GSK3 activation may enhance the sensitivity of mPTP opening and increase the probability of tumor regression [13].

## 10. Other Targeting Candidates for mPTP Opening

Cyt C has been considered “a point of no return” causing apoptotic cellular death [6]. mPTP is the only gate-regulating translocation of Cyt C from the mitochondrial inner membrane space to cytosol. Most importantly, Cyt C release is regarded as a critical step of mitochondria-mediated apoptotic cascade. Accordingly, modulation of mPTP function/opening is more focused on the release of Cyt C from mitochondria and could lead to an opportunity for competent and selective anticancer therapy.

## 11. Pharmacological Switches of mPTP Opening/Closing in Cancer Therapeutics

### 11.1. Modulator/Antagonist of Bcl-2 Family Proteins

A novel range of anticancer molecules has developed with the progress of BH3-mimetic agents. Such new agents and/or compounds have been designed in such a way to selectively regulate or kill cancer cells by targeting the cellular machinery involved in their survival [95]. These developed agents can induce apoptosis by mimicking the action of natural negative switches of Bcl-2 and other related proteins [96]. There is an investigation wherein the concept was utilized with selective inhibitors targeting the different antiapoptotic Bcl-2 family members expressed at the mitochondrial level to determine if they influence mitochondrial matrix Ca^2+^ retention capacity and mPTP sensitivity. Bcl-2, Bcl-xL, and Mcl-1 have been reported to play a key role for mPTP opening. In addition, Mcl-1 is mainly located at the mitochondrial outer membrane, the inner membrane, and the matrix that may strengthen Mcl-1 to execute mPTP sensitivity as compared to the other members localized on to the outer membrane. It has been suggested that the inhibition of each antiapoptotic Bcl-2 family member could lead to decreased mitochondrial calcium retention capacity due to the sensitization of mPTP opening.

ABT-737 is the foremost developed agent designed by Abbott Laboratories (North Chicago, IL, USA) and has been considered the prototype of BH3 mimetics, as it was the ‘first line’ compound established to mimic the function of BH3-only proteins [97]. It has been studied that the obstinacy of several kinds of cancer cells to ABT-737 may be due to ABT-737′s inability to target another prosurvival protein, Mcl-1. The predicted influence of targeting such protein mechanisms of apoptosis resistance was investigated in several kinds of cancers [98,99]. Notably, ABT-737 could act as an apoptotic executioner synergistically with chemotherapeutic molecules to overcome the chemoresistance and in particular kill cancer cells without causing severe toxicity [100,101]. Furthermore, ABT-737 has also been reported to sensitize radiation and chemotherapeutic agents including etoposide [102], doxorubicin [103], and cisplatin [104,105] in different cancer cell lines.

**Figure 4:**
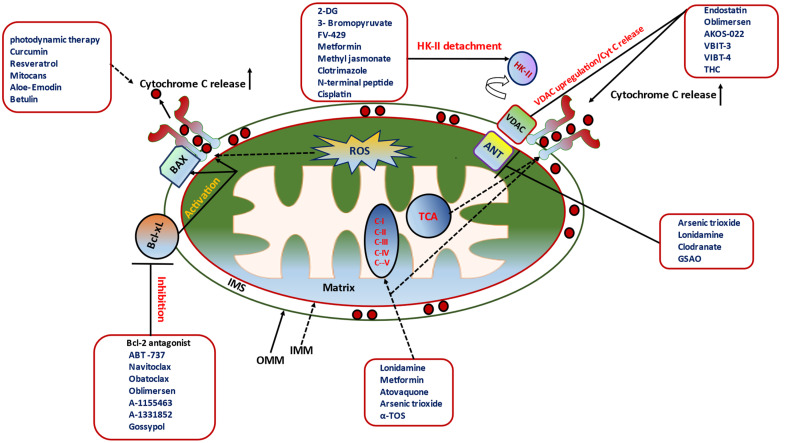
Pharmacologically targeting mPTP-mediated cancer cell death signaling in mitochondrion using chemotherapeutic agents and natural compounds.

ABT-263, an orally bioavailable derivative of ABT-263, is a potent Bcl-2 selective inhibitor [106] (Figure 4). ABT-263 has been shown as an in vitro anticancer activity against a panel of cancer cell lines, either as single agents or in combination with chemotherapeutics [107,108]. In addition, in vivo experiments showed that this Bcl-2 inhibitor could also induce rapid and complete tumor suppression in multiple xenograft models established using small-cell lung cancer [109,110] and hematologic cell lines [111].

To overcome the off-target effect and toxicity linked with Bcl-2 inhibition, Venetoclax (ABT-199), developed by Abbvie, demonstrated a great robustness of anticancer activity against several kinds of hematological malignancies [112,113]. This US FDA-approved drug was employed for the therapeutic intervention of chronic lymphocytic leukemia and small lymphocytic lymphoma as a single therapy and in combination with azacitidine [114,115], decitabine, or low-dose cytarabine [116,117].

In addition, gossypol, a natural phenolic compound found in cotton plants, has been depicted as inhibiting Bcl-2, Bcl-xL, Bcl-W, and Mcl-1 in various cancers, including prostate cancer, breast cancer, leukemia, and gliobastoma [118,119,120,121,122]. Likewise, Gx15-070 (obatoclax), established as a small molecule indole bipyrrole agent, could show significant inhibition of Bcl-2, Bcl-xL, Bcl-W, and Mcl-1 [123]. This inhibitor was developed and investigated to efficiently disrupt the interaction between BAK and Mcl-1 in multiple myeloma [123,124] (Figure 4).

Furthermore, the organic compound HA14-1 was put forward in as to pointedly inhibit Bcl-2 [124,125]. However, HA14-1 is reported to enhance the sensitivity of cancer cells to chemotherapy and radiotherapy [126,127,128]. In the context of molecular signature, oblimersen, an 18-mer phosphorothioate Bcl-2 mRNA antisense oligonucleotides, has the capacity to anneal the initiation codon region of Bcl-2 mRNA, thereby inhibiting Bcl-2 biosynthesis [129]. This mRNA was also studied to directly bind to VDAC, decreasing the channel conductance of VDAC [130]. AZD5991, a selective BH3-mimetic, was recently investigated in clinical trials and showed significant inhibition of Mcl-1 in murine lymphoma models and resulted in a prolonged survival of Mcl-expressing PDX mice [131]. BH3 mimetic antagonists of other antiapoptotic proteins have also been developed as A-1155463 and A-1331852 for Bcl-xL or S63845, AMG 176, for Mcl-1 [131,132,133] in cancer (Figure 4). The overall discussed inhibitors of Bcl-2 have been shown to precisely induce apoptosis in malignant cells and have been greatly demonstrated as single agents and in combination with other drugs in several malignancies, including acute leukemia, lymphomas, and solid tumors (Table 1).

### 11.2. Pharmacological Disruptors of HKII–VDAC Interaction

In the context of glucose analogues employed to target HKII, some key modulators such as 2-deoxy-D-glucose (2-DG) and 3-Bromopyruvate (3-BPA) were reported in previous studies [70,134]. 2-DG administration has been shown a competence with glucose for its entrance into the cell, and once it is penetrated into the cell, it is phosphorylated by HKII to form 2-deoxyglucosephosphate (2-DG-P), which further blocks the glycolysis [70]. 2-DG has been under clinical trials (Phase I/II) to test its efficacies in advanced and hormone refractory prostate cancer (Table 1). It has been shown that a cellular gain of 2-DG-P could also inhibit of HKII [135,136]. The HKII attachment to mitochondria is a crucial step for the initiation of apoptotic cascade. Combined formulations of metformin and 2-DG collapsed ATP in a synergistic way and was observed with a considerable combined therapy in pancreatic cancer cells. In addition to its antimetabolic action, some preclinical investigations have reported that 2-DG possesses an additional anticancer efficacy comprising an antiangiogenic role [137,138,139] for blocking cancer metastasis. 2-DG also showed an improved efficacy in autophagy inhibition [140,141,142]. Nevertheless, in vitro and in vivo preclinical investigations suggested possible synergistic therapeutics in combination with conventional chemotherapy, including cisplatin [143,144] and doxorubicin [145,146]. Convincing data have also been displayed in combination with radiotherapy for the treatment of glioblastoma [147,148]. By combining with mitochondria-targeted carboxy-proxyl (Mito-CP), 2-DG has also demonstrated significant tumor degeneration, advocating that a dual focus of mitochondrial bioenergetics metabolism and glycolytic inhibition may represent a promising chemotherapeutic paradigm [149].

3-BPA is also highlighted as an alkylating agent that tends to covalently modulate sulphydryl groups of HKII. Such kinds of chemical alteration could lead to deterioration of HKII from mitochondria, which provokes apoptosis and further induces cellular destruction [150]. In addition, FV-429 has been recognized as a synthetic flavone that has a potential action to trigger apoptosis in cancer cells. Specifically, FV-429 disrupts the interaction between HKII and VDAC, thereby inhibiting glycolysis-inducing mitochondrial-mediated cell apoptosis [85] (Table 1). An antidiabetic drug, Metformin, could also suppress different kinds of cancers [151,152,153]. Previous reports suggested that metformin could inhibit HKII in lung carcinoma cells, leading to minimizing the glucose uptake and phosphorylation of glucose [154].

Cancer cells are characterized by enhanced glycolytic activity and decreased mitochondrial oxidation, regardless of oxygen availability (i.e., Warburg effect). The overstimulation of glycolysis causes lactate overproduction, triggering a condition of metabolic acidosis in tumor microenvironment [155]. Dichloroacetate (DCA), an orally available small molecule, prevents the metabolic acidosis condition in tumor microenvironment [155,156]. Specifically, DCA inhibits pyruvate dehydrogenase kinase (PDK), selectively targeting cancer cells and switching their metabolism from glycolysis to oxidative phosphorylation. The enhanced pyruvate flux into mitochondria further triggers mPTP activation, increasing efflux of Cyt C and other apoptotic-inducing factors and overproduction of ROS, subsequently reducing cancer cell viability [156].

Bao et al. have observed that the steroid derivatives extracted from *Ganoderma sinense*, such as (22E, 24R)-6β-methoxyergosta-7,9, 22-triene-3β and 5α-diol, demonstrated a high HKII binding affinity [157] (Figure 4). This was the foremost nutraceutical that is reported to show HKII inhibitory action for cancer therapy [157,158]. Another natural compound, methyl jasmonate, also exhibits an excellent binding affinity with HKII [157]. The high affinity of methyl jasmonate with HKII causes dissociation of HKII from VDAC1, causing mPTP opening, which further promotes cytochrome c release and caspase-mediated cell apoptosis [159,160]. In addition, clotrimazole displays its capacity to induce HKII detachment from mitochondria in different cancer cells and efficiently triggered a dose dependent cell apoptosis [74,161]. Nevertheless, the toxic profile of clotrimazole on mitochondria emerged as an option for interfering mitochondrial respiration by promptly binding to a molecular target other than HKII [162] (Figure 4) (Table 1). In addition, several compounds have already been investigated for their capacity to either directly interact or alter the activity of VDAC [163,164]. Erastin binds to VDAC and disrupts the HKII–VDAC interaction, triggering the opening of mPTP and subsequently inducing cell apoptosis [163,164]. In addition, furanonaphthoquinones and oblimersen could be able to interact with VDAC1 and prevent or inhibit the VDAC1 oligomerization and thereby cause mitochondrial dysfunction and apoptosis, and thus are considered as novel anticancer agents that facilitate the regulation of VDAC.

Shulga et al. have highlighted the importance of HKII–VDAC interaction as one the most prominent components regulating the apoptotic signaling in cells [165]. This research group has synthesized an N-terminal peptide of HKII (15 amino acid residues) that targets VDAC. The therapeutic setup of N-terminal peptide of HKII resulted in dissociation of HKII from the mitochondria to the cytosol. It was also suggested that the dissociation of HKII from the mitochondria may sensitize the mitochondria to the cisplatin-triggered cellular impairment, causing an increased cytotoxicity [165].

### 11.3. Pharmacological Inhibition of ANT

ANT is considered an excellent target of arsenic trioxide within the mPTP hub. Arsenic trioxide is well proven as a potent agent against acute promyelocytic leukemia [166]. In the context of proapoptotic execution, one of the key mechanisms was established with an apoptotic effect via the opening of the mPTP [167]. Arsenic trioxide was also considered as an agent disrupting mitochondrial membrane potential, and the proapoptotic effect triggered by arsenic trioxide could also be due to its ability to inhibit Bcl-2 [168]. Likewise, Lonidamine (LND) was developed as an indazole carboxylate and has been investigated to target ANT to induce mitochondria-mediated apoptotic cell death [169] (Figure 4). Instead of its cytostatic potential in recurrent glioblastoma multiforme and further improvement of response rate in epirubicin therapy for advanced breast cancer patients, LND was determined to be as toxic to nontumor tissues and was shown to cause hepatotoxicity [170]. Nevertheless, LND has also been considered in clinical trials with the supplementation of chemotherapeutics in several cancers [171,172]. For instance, LND has been reported with limited efficacy tested in lung cancers in both Phase II and III trials [173,174]. In addition, clodranate (a nitrogen-free bisphosphonate) exhibits a competitive inhibition in ANT, causing the impairment of the mitochondrial membrane potential and leading to apoptotic cell death [169]. However, the underlying mechanisms are still unclear. It was also observed that the administration of clodronate to postoperative adjuvant therapy has shown considerable improvement in overall survival among breast cancer patients [175]. Furthermore, GSAO (4-(N-(S-glutathionylacetyl)amino) phenylarsenoxide) is a well-recognized cross-linker to cysteine residues of ANT and showed an inhibition in ATP/ADP exchange via ANT, causing depolarization in the mitochondrial membrane and inducing apoptosis [176] (Figure 4). Interestingly, GSAO is established to be a more promising anticancer drug due to its inhibitory activity on tumor angiogenesis by targeting mitochondria in proliferating endothelial cells [176] (Table 1).

### 11.4. Pharmacological Triggers of Cyt C Release in Cancer Cell Death

It has been implicated that apoptotic sensors are actively able to trigger Cyt C release that could be employed as a powerful alarm to induce apoptotic cancer cell death [177,178]. Several reports have hypothesized that photodynamic therapy (PDT) is more capable of causing cancer cellular damage by altering mitochondrial membrane potential, thereby stimulating the release of Cyt C and activating other caspase-dependent apoptotic cellular death [179,180]. There is an important recognition for distinct photosensitizers applied in PDT, which was considerably approved for clinical utilization and/or is still under clinical trials [181,182]. In the context of nutraceutical therapy, several compounds have been developed to induce apoptosis-mediated cancer cell death and has displayed promising data of particular interest. Likewise, resveratrol [183,184], curcumin [185], aloeemodin [186], and betulin [187] were investigated for trigger apoptosis in several kinds of cancer cell lines via enhancing the release of Cyt C from mPTP to cytosol (Table 1). Resveratrol has been clinically tested under Phase I trials with colon cancer patients. There are some promising clinical trials for using the FDA-approved curcumin for chronic myeloid leukemia, multiple myeloma, and prostate, colorectal, and pancreatic cancer patients [188]. Other compounds, such as mitocans, have been used to target mitochondria and induce apoptosis in breast, colorectal, small-cell lung, and prostate cancer [189,190].

### 11.5. Pharmacological Modulation of ETC in Cancer Cell Death

ETC complexes act as an entrance gate of electrons in the ETC and are capable of regulating the mitochondrial ATP synthesis by pumping protons toward the intermembrane space. It has been evident that such complexes participate in tumor formation and trigger metastasis by elevating ROS contents. Interestingly, it has been established that complex modulators could alter mPTP architecture. ETC complexes directly interact with mPTP and regulate the mPTP closing/opening status. In addition, cancer cells were placed in a cross talk between the complex junction and mPTP to achieve cellular death and/or regulate the complex activity. ETC has also been considered as a major culprit in apoptosis in cancer cells. Indeed, metformin has been clinically tested in different phases and trials for cancer patients [191]. So far, extensive data has been reflected toward Phase III clinical trial wherein the potential of metformin was tested for the therapeutic manifestation of breast cancer, low-grade malignant cancers, and other benign cancers [192]. Metformin was shown as a key modulator for the Complex I platform in mitochondria-mediated apoptosis. In contrast to Complex I, no well-developed Complex II inhibitor was discussed at the biomedical hub (Figure 4). Nevertheless, LND has been placed in a research pharmacological rack and shown to hinder Complex II in isolated mitochondria and in DB-1 melanoma cells [193,194,195]. Atovaquone is another FDA-approved molecule considered to target pneumocystis, pneumonia, and malaria [196]. This drug has been known as a ubiquinone analogue that shows an inhibition of Complex III in cancer cell lines, reducing the mitochondrial oxygen consumption rate and diminishing the hypoxia induced in cancer with considerable doses [197,198,199,200,201]. In addition, arsenic trioxide has been well documented as a Complex IV inhibitor and is a FDA-approved compound for targeting acute promyelocytic leukemia [166]. One of the crucial mechanisms of the arsenic trioxide-mediated apoptosis is through the opening of the mPTP [166,202] (Figure 4). For the past few years, arsenic trioxide-mediated apoptosis has been well demonstrated as mPTP and other mitochondrial-mediated apoptotic cell death in several kinds of cancers [203,204,205,206]. At the preclinical stage, this mechanism was shown as a great inhibitor for mitochondria-mediated cellular respiration and exhibited as minimizing hypoxia in lung carcinoma (Table 1).

### 11.6. Nutraceuticals (Natural Compounds): Excellent Armor for Apoptotic Cancer Cell Death

Nutraceuticals have been known to minimize the level of antiapoptotic signals in cancer cells. Resveratrol is a polyphenolic agent extracted from grapes and wine and has been shown to be able to inhibit mitochondrial ATP synthesis and thereby trigger MOMP [169]. One of the synthetic resveratrol analogues, HS-1793, was screened to disrupt the mitochondrial membrane potential, subsequently promoting release of Cyt C and triggering the opening of mPTP in murine breast cancer cells [207]. Additionally, this analogue was also shown to enhance Bcl-2-mediated antiapoptosis in leukemia cells [208]. Resveratrol has been considered in a few clinical trials in cancer patients [209] (Table 1). In recent years, 4-amido-2,4-pentadieneoate (APD)-class peptide was prepared from a bacterial extract (*Nocardiopsis sp.*) from marine mollusks. Natural contents of such an APD-class have been established as hypoxia-triggered cytotoxins, targeting mitochondria [94]. On the other hand, cernumidine (50) is a well renowned guanidinic alkaloid that exhibits anticancer efficacy via the downregulation of Bcl-2/BAX ratio, leading to loss of mitochondrial membrane permeability with synergistically treated cisplatin in T24 cells [210]. Lycorine (51) was extracted from the Amaryllidaceae family and could cause the mPTP opening and mitochondrial Ca^2+^ and Cyt C release and ultimately induce apoptosis in HepG2 cells [211]. Likewise, amorfrutin C (73) is another nutraceutical armor that belongs to the amorfrutin benzoic acid class of compounds found in *Glycyrrhiza foetida*. The therapeutic regime using amorfrutin C shows considerable disruption of mitochondrial integrity, which results in an irreversible opening of mPTP in HT-29 cells [44]. There is increasing recognition of other nutraceuticals such as allyl isothiocyanate [212], α-conidendrin [213], dehydrobruceine B [214], frugoside [215], methyl caffeate [216], tetrahydrocurcumin [217], phloretin [218], and sesamol [219] for their anticancer capacities. These nutraceuticals demonstrate anticancer capacities through disruption of mitochondria through ROS production, Cyt C release, loss of mitochondrial membrane potential, and modulation of Bcl-2 family members, ultimately leading to mPTP opening and inducing cell apoptosis. In recent decades, curcumin has been extensively employed as a major constituent of turmeric powder from the plant *Curcuma longa*. In the context of its anticancer potential, curcumin is established as an excellent modulator of Bcl-2 family proteins and cellular ROS generation. Correspondingly, betulinic acid, a natural pentacyclic triterpenoid, has been known to stimulate mitochondria-mediated apoptosis in cancer cells [169]. This compound not only enhances MOMP but also inhibits Bcl-2 family proteins in cancer [169,220] (Table 1). Alkaloids, such as phyto-compounds, have been well investigated for their use in inhibiting proliferation and metastasis in cancer cells. Berberine (an alkaloid), derived from the Berberidaceae plant family, exhibits potency either directly on mitochondria or selectively with a possible interaction of ANT at the mPTP juncture [221]. Several other cellular machineries underlying apoptotic induction were triggered by berberine through its modulation on ROS generation and reduction in mitochondrial membrane potential, leading to mPTP opening, the irreversible release of Cyt C, and alteration in the Bcl-2/BAX ratio [169]. In addition, pre- and postsupplementation of vitamins has also played a crucial role in cancer cell impairment. For example, α-Tocopheryl succinate (α-TOS) has been known as a vitamin E analogue competitively bound with ubiquinone to specific Q sites at ETC Complex II. The α-TOS/ETC Complex II binding subsequently leads to the detachment/destabilization of ubiquinone from Complex II, disruption of electron flux, and subsequently overproduction of ROS [222]. It has been known that ROS is more capable of catalyzing the construction of disulfide bridges between the monomers of BAX in the cytosolic compartment, leading to a conformational alteration for dimerization or forming channels by translocated BAX in the outer mitochondrial membrane [223] (Figure 4). Likewise, one hypothetical scheme was also proved with α-TOS-induced apoptosis that could involve the Noxa-Bak axis [224]. Polyphenols, isolated from herbal species, have been demonstrated with promising anticancer activities. For instance, honokiol is a well-established polyphenolic molecule extracted from the genus Magnolia [225]. In recent years, both antiangiogenic and antitumor activities of honokiol have been investigated in preclinical studies. It has been suggested that honokiol can induce CyP-D and promote mPTP opening. Notably, the honokiol-induced CyP-D expression further impaired Bcl-2 and Bcl-xL balance, triggering apoptosis in the resistant leukemia cells [226].

**Table 1:** Overview of the clinical trials of drugs targeting mPTP-mediated apoptotic cascade in cancers.

Compounds/Inhibitors	Targets	Preclinical/Clinical Status Clinical Trial.gov Identifier/Reference	
Bcl-2 Modulators/Inhibitors			
ABT737	Bcl-2 family	Preclinical	[97,100,101]
ABT199	Bcl-2 family	Phase II	NCT01889186 [112,113]
Gossypol	Bcl-2 family	Preclinical	[118,121]
Obatoclax	Bcl-2 family & Mcl-1	Preclinical	[122]
AZD5991	Mcl-1	Phase I	NCT03218683
AMG176	Mcl-1	Phase I	NCT02675452
S63845	Mcl-1	Phase I	NCT02992483
HKII -VDAC-Mediated Metabolic Inhibitors			
3-Bromopyruvate	HKII–VDAC interaction	Preclinical	[70]
2-DG	HKII–DAC interaction	Preclinical Phase I/III	NCT00096707, [135,136]
FV-429	HKII–AkT	Preclinical	[85]
Methyl Jasonate	HKII–VDAC interaction	Preclinical	[159,160]
Clotrimazole	HKII–VDAC interaction	Preclinical	[74,161]
Erastin	HKII–VDAC interaction	Preclinical	[164]
ANT Inhibitor			
Arsenite	ANT proteoliposomes	Preclinical	[168]
Lonidamine	ANT	Preclinical	[171,172,173,174]
Clodranate	ANT	Preclinical	[169,175]
GSAO	ADP/ATP exchange via ANT	Preclinical	[176]
Cytochrome C Sensitizer			
Photodynamic therapy	Cyt C	Phase III	NCT02064673, [180,181]
Resveratrol	Cyt C	Preclinical	[183,184]
Curcumin	Cyt C	Phase II	NCT02944578/NCT02782949
Aloe-Emodin	Cyt C	Preclinical	[186]
Betulin	Cyt C	Preclinical	[187]
ETC Modulators			
Metformin	Complex I	Phase III	NCT01101438, [192]
Lonidamine	Complex II	Phase II/III	NCT00237536/NCT00435448
Atovaquone	Complex III	Phase I/Preclinical	NCT02628080, [195,196,197,198,199,200]
Arsenic Trioxide	Complex IV	Preclinical	[166,203,204,205,206]
Nutraceuticals on mPTP Modulation			
Resveratrol (analogue, HS-1793)	Cyt C/Bcl-2	Preclinical	[207,208,209]
Cernumidine	Bcl-2/BAX ratio	Preclinical	[210]
Lycorine	mitochondrial Ca^2+^	Preclinical	[211]
Amorfrutin C	ETC	Preclinical	[44]
Isothiocyanate	Cyt C	Preclinical	[212]
α-conidendrin	Bcl-2	Preclinical	[213]
Dehydrobruceine B	Cyt C	Preclinical	[214]
Frugoside	Cyt C	Preclinical	[215]
Methyl caffeate	Bcl-2	Preclinical	[216]
Phloretin	ETC	Preclinical	[218]
Sesamol	ETC	Preclinical	[219]
Betulinic acid	Bcl-2	Preclinical	[220]
Berberine	ANT	Preclinical	[221]
α-TOS	Complex II	Preclinical	[222,223]
Honokiol	CyP-D	Preclinical	[164,225]

## 12. Preclinical and Clinical Models for mPTP-Mediated Cancer Cell Death

In recent years, several studies were published to discuss VDAC-binding compounds and/or agents [164] and their functions on regulating mPTP status in drug-treated colorectal cancer cells. In addition, mitochondrial immunoprecipitation (Mito-IP) assay demonstrated that ANT-1 and Cyp-D formed a complex in erastin-treated HT29 colorectal cells and is considered as a window of opportunity for induction of mPTP opening; the level of cytosolic Cyt C was also increased in HT-29 cells, inducing opening of the mPTP junction [164]. Several pharmacological mPTP blockers, including sanglifehrin A [227,228], were applied to modulate mPTP in cancer cells. Zhang et al. (2018) have suggested that hirsutine, a nutraceutical, could potentially provoke mitochondrial impairment and induce caspase-dependent apoptotic cascade, enhancing the opening of mPTP, in human lung cancer cells. In addition, hirsutine promotes the dephosphorylation of GSK3β the depletion of ATP and facilitates mPTP-mediated cell apoptosis in cancer cells [229]. In 2021, Zhang et al. observed that Cr(VI) exposure could cause Ca^2+^ elevation and activate the Ca^2+^-associated signaling mechanism, which is also a main culprit for an extensive opening of mPTP, leading to activation of apoptotic cascade in liver cancer (Hep3B) cells [230]. It has been well noted that in the resting state, CypD stays in the mitochondrial matrix to regulate mPTP closure. Nevertheless, CypD also connects with ANT at the inner mitochondrial membrane to open the mPTP if masking is under crucial conditions [231]. One report has demonstrated that CsA could effectively modulate apoptosis, advocating the cytotoxic potential of esculetin critically linked with CypD, and it was highlighted as a key player in esculetin-induced cell death. This observation was also verified by silencing CypD in human gastric cancer (SGC-7901, MGC-803, BGC-823) cells [232]. One interesting study showed that ABT-737 and curcumin significantly suppress melanoma (WM-115 and B16) cells through a massive induction of cell apoptosis. Furthermore, combining curcumin with ABT-737 synergistically provoked mPTP opening in melanoma cells [233]. Recently, Xue et al. (2021) found that accumulation of Ca^2+^ in mitochondria would lead to mitochondrial ROS accumulation, ΔΨm depolarization, mPTP opening, and ultimately cellular death and/or apoptosis established in breast cancer cell lines MDA-MB-231, MCF-7, and SKBR-3 [234,235].

Moreover, the isolated proteins and reconstituted liposomes established on the VDAC at the OMM and the ANT at the IMM were extensively studied [236,237]. Interestingly, knocking down VDAC2 using siRNA showed enhanced sensitivity to altering mPT in VDAC1^−/−^ and VDAC3^−/−^ mice [236]. In addition, HKII functions as a key component in the mPTP complex and is involved in HKII-mediated tumorigenesis in multiple cancer types [238,239]. Sun and Zhang suggested that miR-143 targets HKII, which suppresses oral squamous cell carcinoma (OSCC) cell growth, invasion, and glucose metabolism in both in vitro and in vivo systems [238,239,240].

## 13. Concluding Remarks

In conclusion, this review aims to focus on mitochondrial function in regulating cellular survival/death switching and how mitochondrial dysfunctions execute the translocation of cytochrome c for initiating apoptotic cell death in cancer episodes. Accumulating evidence highlights mPTP as the major gate for the cytochrome c release, and thereby causing apoptotic cancer cell death. We also discussed disruption of MOMP as a more promising possibility that could activate/stimulate upstream proapoptotic signaling cascade that is intermittently deregulated and more resistant to chemotherapeutic agents in cancers. As described in this review, modulating mPTP opening, via small molecules/compounds and natural products, has proven to be a promising target-specific therapeutic regimen in treating cancers. Understanding mPTP structure and functional mechanisms will provide molecular insight and facilitate the development of novel approaches toward improving the quantity of precision medicine in cancers. Further investigation is also summoned to explore the detailed mechanism of MOMP via the mPTP hub in cancers.

We have clarified in this review that permeability of the mitochondrial membranes is a decisive location wherein cancer cellular survival or death could be determined. Moreover, a deeper understanding of the molecular mechanisms underlying mPTP-mediated apoptotic cancer cell death would pave a new path for developing novel therapeutic strategies against cancers.
